# Intuitive Participation in Aggressive Intergroup Conflict: Evidence of Weak Versus Strong Parochial Altruism

**DOI:** 10.3389/fpsyg.2016.01535

**Published:** 2016-10-05

**Authors:** Robert Böhm

**Affiliations:** Decision Analysis, School of Business and Economics, RWTH Aachen UniversityAachen, Germany

**Keywords:** intergroup conflict, parochial altruism, dual systems decision making, team games, ego depletion

Humans are unusually cooperative and prosocial, sharing resources with kin and non-kin others. At the same time, they engage in violent intergroup conflict and discriminate against members of other groups. How can we explain this apparent inconsistency? Building on Darwin (1871), it has been proposed that self-sacrificing prosociality toward the in-group and hostility toward the out-group may have co-evolved (Choi and Bowles, 2007; García and van den Bergh, 2011). Research on so-called *parochial altruism*, i.e., the motivation to benefit in-group members at personal cost, while not benefitting or even harming out-group members, recently received much attention in psychology and beyond (for reviews see, De Dreu et al., 2014; Rusch, 2014; Yamagishi and Mifune, 2016). Empirical studies aiming to support the parochial altruism hypothesis yielded mixed results, though. For instance, whereas some studies provided support for the parochial altruism hypothesis (e.g., Bernhard et al., 2006; Abbink et al., 2012), others failed to show a relationship between individual-level prosociality and intergroup discrimination (e.g., Corr et al., 2015). And yet others found that prosocial individuals are less likely to discriminate against out-group members (e.g., Thielmann and Böhm, in press).

De Dreu et al. (2015) provide a novel perspective to this debate by showing that parochial altruistic behavior increases in intuitive compared to deliberative decision making. The paper is among the first to combine research on intuitive vs. deliberative decision making (e.g., Rand et al., 2012, 2014; Bear and Rand, 2016) with research on the participation in destructive intergroup conflict (e.g., Bornstein, 2003; Choi and Bowles, 2007; Rusch, 2014). In detail, the authors used an easy vs. difficult Stroop Interference Task (Stroop, 1935) to manipulate cognitive self-control prior to an intergroup conflict game. In this game—the Intergroup Prisoner's Dilemma—Maximizing Difference (IPD-MD; Halevy et al., 2008)—participants could decide between selfishly keeping their endowment or contributing it to one (or both) of two public goods. Either they contributed to a within-group pool that benefits their in-group without affecting the out-group. Alternatively, they could have also contributed to a between-group pool that equally benefits the in-group as the within-group pool, but additionally harms the out-group. Because the between-group pool decreases the outcome of the out-group absolutely and relatively to the other options available, contributions to this pool can be interpreted as parochial altruism. Although the contributions to the between-group pool were generally low as in previous research (e.g., Halevy et al., 2008, 2012; De Dreu et al., 2010; Weisel, 2015; Weisel and Böhm, 2015), De Dreu et al. (2015) found that they increased when participants were cognitively taxed. This effect was independent of participants' social value orientation, i.e., their general preference for the welfare of others (Van Lange and Kuhlman, 1994; for an overview see, Murphy and Ackermann, 2014). The findings imply that parochial altruism might be more strongly pronounced when individual self-control is low. Furthermore, they can be interpreted as support of the claim that prosociality toward the in-group and parochialism toward the out-group may have co-evolved. In particular, one could argue that intuitive behavioral tendencies of benefitting in-group members while—at the same time—harming out-group members are based on adaptive evolutionary processes, but are inhibited because modern intergroup relations are less violent (Pinker, 2011). The study might therefore be the starting point of a novel research program combining (different) manipulations of intuitive vs. deliberative decision making with intergroup social dilemmas.

The insights of De Dreu et al. (2015) contribute to the recent debate on the specific circumstances under which people do (not) show parochial altruistic behavior, as apparent from the numerous papers that appeared in the Frontiers Research Topic *Parochial altruism: Pitfalls and prospects* (Rusch et al., 2016). Here, I would like to point to a conceptual ambiguity that may have important consequences for the interpretation of the findings by De Dreu et al. (2015) and others: In the IPD-MD it is not only the absolute amount but also—and in particular—the relative amount of tokens contributed to the between-group pool *compared with* the within-group pool that is crucial for the interpretation. In detail, a larger contribution to the between-group pool relative to the within-group pool may be interpreted as negative attitude toward the out-group, an equal contribution to both pools as indifference regarding the out-group's welfare, and a smaller contribution to the between-group pool as positive attitude toward the out-group. Importantly, although De Dreu et al. (2015) show differences in contributions to the between-group pool between experimental conditions, the contributions to the within-group pool are always exceeding those to the between-group pool. As such, their findings indicate that in the high taxing condition, the attitude (and the behavior resulting from it) toward the out-group is *less positive* rather than *more negative* compared to the low taxing condition. And indeed, both effects are consistent with the interpretation of increased parochial altruism given the definition provided above (“[…] not benefitting *or* even harming members of other groups […]”; italics added). Therefore, in order to increase the conceptual clarity of in-group-bounded prosociality, I propose a semantic differentiation between effects that are based on a lack of positive attitudes toward the out-group, i.e., *weak parochial altruism*, and effects that are due to negative attitudes toward the out-group, i.e., *strong parochial altruism*. Although the attitudes toward the out-group are on a positive-negative continuum, I suggest that the resulting discriminatory behaviors constitute different psychological qualities and, furthermore, that the transition from weak to strong parochial altruism might follow specific requirements and processes. Future research should show the joint and differential predictors of weak vs. strong parochial altruism, e.g., by classifying participants into different types (i.e., weak vs. strong parochial altruists) based on their relative contribution behavior. Combining novel tests on mediating and moderating factors as well as a more fine-grained conceptualization of parochial altruism effects itself could eventually advance our understanding of intergroup conflict in general.

## Author contributions

The author confirms being the sole contributor of this work and approved it for publication.

### Conflict of interest statement

The author declares that the research was conducted in the absence of any commercial or financial relationships that could be construed as a potential conflict of interest.
